# Mother's and Father's Migrating in China: Differing Relations to Mental Health and Risk Behaviors Among Left-Behind Children

**DOI:** 10.3389/fpubh.2022.894741

**Published:** 2022-06-03

**Authors:** Hailati Akezhuoli, Jingjing Lu, Guanlan Zhao, Jiayao Xu, Menmen Wang, Feng Wang, Lu Li, Xudong Zhou

**Affiliations:** ^1^Institute of Social Medicine, School of Medicine, Zhejiang University, Hangzhou, China; ^2^School of Public Health, Hangzhou Normal University, Hangzhou, China; ^3^Zhejiang Shuren University, Hangzhou, China

**Keywords:** left-behind children, migrating mother, migrating father, previous left-behind, mental health, substance use, internet addiction

## Abstract

**Background:**

In China, the figure for left-behind children (LBC) of migrants stood at 68. 77 million in 2015. Despite being seen as a whole in the last few decades, LBC today differ broadly in parental migrating status. This study focused on LBC with both parents migrating (BLBC), LBC with only mothers migrating (MLBC), LBC with only fathers migrating (FLBC), and previous LBC with one or both parents migrating (PLBC), separately. We aimed at exploring the extent to which LBC were being affected by each migrant parent on both mental health and risk behaviors.

**Methods:**

Data from 4,832 children were collected by a school-based survey in both rural and urban areas of China's Anhui province. Each participant anonymously completed a self-administered questionnaire containing the sociodemographics, the Strength and Difficulties Questionnaire (SDQ), the items from the Youth Risk Behavior Surveillance System (YRBSS), and Young's Internet Addiction Test for Chinese (YIAT-C). Data were analyzed using one-way ANOVA and the Chi-squared test. Associations were estimated by multiple linear regression and logistic regression analyses adjusted for several confounders.

**Results:**

The results suggested that BLBC (*p* < 0.001), MLBC (*p* < 0.05), FLBC (*p* < 0.01), and PLBC (*p* < 0.001) significantly scored higher for total difficulties along with emotional symptoms and conduct problems than never left-behind children (NLBC). Besides, BLBC, FLBC, and PLBC further reported a significantly higher rate of smoking (*p* < 0.001, *p* < 0.01, and *p* < 0.001, respectively) and drinking (*p* < 0.01, *p* < 0.05, and *p* < 0.01, respectively) than did NLBC. Also, MLBC appeared higher risks of smoking problems [OR = 2.31, 95% CI (1.45–3.69), *p* < 0.001] and the internet addiction [OR = 2.15, 95% CI (1.24–3.72), *p* < 0.01], when compared to NLBC.

**Conclusions:**

The findings provided insight into LBC within the different contexts of parental migrations and contributed to a better understanding of their specific and potentially persistent health risks. Correspondingly, the study highlighted the implications for differentiating LBC to capture the more vulnerable group and tailored interventions to prioritize.

## Introduction

In recent times, China is seeing an ever-burgeoning process of urbanization and industrialization. Accordingly, there is a colossal internal migration where the rural laborers are heading to cities for better-paid jobs. However, the migrants are limited by the rigid system of household registration (the hukou system) to gain urban services, from access to housing, health care, and education to compensation payouts (1). All these services, together with high living and education expenses in cities, leave many migrant parents with little choice but to leave children behind at home and entrust extended family members (often grandparents) to child care. Accordingly, a catchphrase “left-behind children (LBC)” is generated, epitomizing those children “who live in their original domicile, but do not live together with their parents, as either one parent or both parents have migrated” (2). By one estimate, the figure for LBC in China stood at 68.77 million in 2015, with 40.51 million (58.9%) in rural areas and 28.26 million (41.1%) in urban areas (1). Within this group, broadly, the proportions of LBC with both parents migrating (BLBC), LBC with only fathers migrating (FLBC), and LBC with only mothers migrating (MLBC) were estimated to be 49.0%, 30.6%, and 20.4% in rural areas and to be 43.8%, 31.1%, and 25.1% in urban areas, respectively (2). Still, the number of migrants in China, according to the Seventh National Population Census, is scaling up and has hit a staggering 376 million (3). This upward trend of migration, however, is not specific to China but is mirrored globally. Similarly, the labor migrants in search of employment are increasing specifically in low-income and middle-income countries such as the Philippines, Ghana, and Georgia, either internationally or internally (4).

Targeting the impacts of parental migration on LBC's health, a slew of research has risen over the decades. One systematic review noted that parental absence due to migration was generally unfavorable to the health of LBC with no evidence of benefit in terms of mental health (depressive disorder, anxiety disorder, and conduct disorders), substance use, wasting, and stunting (5). As for the psychological concerns, numerous studies also demonstrated that LBC tended to have a higher level of loneliness (6), lower self-esteem, and self-concept (7, 8) as compared to never left-behind children (NLBC). Also, they were more vulnerable than NLBC to anxiety and depression (9–11) and even more likely to develop suicidal ideation (12, 13) and self-injury (13, 14). Yet, in context of the differing parental migration, the findings on mental health among LBC stay mixed.

Some scholars assumed that BLBC struggled with increased depressive symptoms (5, 10), increased hyperactivity with overall difficulties, and reduced psychological wellbeing (15, 16) than did those left behind by one or neither parent. Others further stressed that MLBC were particularly at greater risk than NLBC for mental problems and decreased wellbeing (17, 18). By contrast, FLBC were found to be insignificantly affected by migrant fathers on their psychological health (19, 20). Besides current LBC, as migrant parents returned, there were also a large group of previous left-behind children (PLBC) who had a left-behind experience with one or both parents absent for over 6 months (21). Concerning the long-term impact of parental migration, researchers found a persistently detrimental mark left on the mental health of PLBC. It was suggested that PLBC were more likely than NLBC to have psychosocial difficulties in terms of emotional problems and peer relationships (21), yet there was also the earlier suggestion that PLBC appeared mentally healthier than NLBC (22). As such, a more nuanced look at the implications of each type of parental migration on children's mental health remains in need.

Furthermore, the greater prevalence of risk behaviors among LBC also has come to light in the wake of growing studies. Compared to NLBC, LBC were at increased risk for tobacco and alcohol use (23, 24) and internet addiction (25). Similarly, the evidence of these behavioral risks varied in different migrant parents. For instance, a recent literature review generalized that BLBC were more likely than those left behind by one or neither parent to engage in smoking and drinking (26). Still, other research suggested that both BLBC and MLBC had a more increased likelihood of smoking than NLBC (27). Paternal migration, rather, was identified to be protective for LBC's smoking in the additional study (28). As for internet addiction, while a growing body of studies indicated that BLBC were at greater risk than NLBC (16, 29, 30), few have examined it among LBC left by different parents. Similarly, the research on health risk behaviors among PLBC is quite lacking.

Although either mental or behavioral consequences of being left behind were well documented, the studies to date centered on comparing children with and without the migrant parent(s), seeing LBC as a whole. The questions remain over whether the migration of fathers, mothers, and both parents could differentially influence a broad range of health outcomes among LBC, and whether the mental and behavioral consequences of parental migration also extend to PLBC. The available evidence to conclude, to our best knowledge, was still limited by the consistency, comparability, and insufficient sample (especially of MLBC) (16). Besides, a whole host of studies merely focused on rural LBC. However, the emerging evidence showed that urban LBC might be at more severe health risk when compared to rural LBC, exhibiting higher mental health difficulties (emotional symptoms and total difficulties) and more drinking problems (23). Given the estimated proportion of urban LBC (41.8%) was relatively considerable as that of the rural ones (58.9%) till 2015, there is a need to assure attention to the inclusion of this population.

In light of all this, this study took different parental migration into account, focusing on BLBC, FLBC, MLBC, and PLBC, separately. We sought to explore the specific linkage between each migrant parent, previous parental migration, and their children's health outcomes concerning mental health and risk behaviors related to smoking, drinking, and internet addiction.

## Methods

### Study Setting and Procedure

Between April 2018 and March 2019, a cross-sectional study using a self-administered questionnaire was carried out among students at schools in rural and urban areas in Wuhu, the second-largest city in Anhui province of China. Following the sample size calculated in our previous work (23), schools in Wuhu were targeted as urban samples. As rural locations, two counties of Wuhu city, Wuwei and Nanling, were selected using random cluster sampling. Then, four townships (two for each county) that lagged economically were randomly chosen for investigation. The registered school roster of each surveyed place was provided by the Education Bureaus in Wuhu, Nanling, and Wuwei after acquiring informed consent. In total, 10 schools in urban areas, comprised of five primary schools and five secondary schools, were randomly selected. Of the four townships, one primary school and one middle school each were included. The Education Bureau further assisted in contacting the 18 targeted schools for the approval of the investigation. To ensure the required literacy capability for having the self-completion questionnaire done, students from 5–8 grades of selected schools were invited. The lower grade (1–4 grades) students who found it difficult to complete the questionnaire in our pre-survey were excluded. We did not invite higher grade (9 grade) students for their busy schoolwork schedules. Before performing the survey, informed consent was obtained from all participants, head teachers, and parents or caregivers (sent by letter through students in advance). Questionnaires were distributed by our research assistants and then were completed by the participants in the classroom settings with teachers absent. Respondents were permitted to inquire to comprehend the questions if necessary. Also, they were informed that their participation was strictly confidential, voluntary, and anonymous and that they could quit at any time point.

### Measurement

#### Socio–Demographic Characteristics

Data collected on participants' sociodemographics involved the following: (1) age; (2) gender; (3) grade; (4) household wealth level measured by a perception-based question “how do you think about your household finances level compared with others in your community?,” with options of “much better off,” “better off,” “the same,” and “poorer and much poor”; (5) parental educational level, which referred to the higher education level of either the father or the mother and was classified into “primary school or lower,” “middle school,” “high school or above”; (6) only-child or not; (7) household registration, or hukou, which was identified by asking “what is your official residence, or hukou status?” with two alternatives, urban and rural.

#### Different Parental Migration and Types of LBC

The types of LBC were determined based on specific parental migration by the question “Does your father (and/or mother) migrate to another place for work, and has he (and/or she) no longer lived with you for more than 6 months?” with options “yes, currently migrates,” “yes, previously migrated,” and “no, never migrates”. Drawing upon the responses, we grouped the children into five groups: (1) BLBC: LBC with both parents migrating; (2) FLBC: LBC with only fathers migrating; (3) MLBC: LBC with only mothers migrating; (4) PLBC: previous LBC with one or both parents migrating; (5) NLBC: never LBC.

#### Mental Health

Mental health was evaluated by applying the student's version of the Strength and Difficulties Questionnaire (SDQ), a commonly used screening instrument developed by Goodman (31). It also has proven reliability and validity in the Chinese setting (32). It contains five dimensions including emotional symptoms, conduct problems, hyperactivity, peer problems, and prosocial behaviors, with Cronbach's alpha values of 0.74, 0.78, 0.72, 0.67, and 0.76 in this study, respectively. Each subscale consists of five items ranked on a 3-point Likert scale (0 = not true, 1 = somewhat true, and 2 = certainly true), and its scores are derived by summing up that of each item, ranging from 0 to 10. Then, the scores of four subscales (excluding the prosocial behaviors) count toward the overall difficulties score within the scope of 0 to 40. A higher score of either subscale (excluding the prosocial behavior) or the total difficulties indicates a higher degree of poor mental condition.

#### Health-Related Risk Behaviors

Smoking and drinking problems were assessed by the items of the Youth Risk Behavior Surveillance System (YRBSS) (33). With reference to our past study (24), we incorporated the following binary questions: (1) Have you ever smoked, with one or two puffs counting? and (2) Have you ever drunk alcohol at least once before? For the assessment of internet addiction, we utilized Young's Internet Addiction Test for Chinese (YIAT-C) (34), a 20-item with a 5-point Likert response scale (ranging from 1 = not at all to 5 = always). A higher total score predicates a greater severity of internet addiction. In the final analysis, its scores were divided into the following two categories: Yes and no, corresponding to 51–100 points and 0–50 points (35). The YIAT-C was also found reliable and validated when applied to the Chinese population (alpha coefficient = 0.917) (36).

### Statical Analysis

The analytical procedure was performed using SPSS 24.0 software. The descriptive statistics were conducted across five groups at the outset. The continuous variables, including age and the scores on SDQ, were described as mean (standard deviation, SD). The categorical variables, including gender, grade, family wealth and parental education level, hukou, only-child status, and risk behaviors outcomes, were analyzed as the percentage. Differences in sociodemographics between groups were tested using ANOVA, the Chi-squared test, and the Kruskal–Wallis test appropriately. Also, ANOVA and the Chi-squared test, followed by the Scheffe's test or the Bonferroni test, were used to compare mental health and behaviors across all groups, respectively. A two-sided *p* of 0.05 was considered a significant level. Multiple linear regression and logistic regression analyses were carried out to evaluate the relevance between different parental migration and the primary outcomes (including psychological scores and prevalence of risk behaviors among children), as appropriate. Models were performed with and without adjustment for sociodemographic confounders (e.g., age and gender) in the univariate analysis. The results were presented as β or odds ratios (ORs) with 95% confidence intervals (CIs).

## Results

Among the 5,393 participants initially included, 140 were excluded for outright refusals or incompletion of key variables (parental migration status). The response rate reached 97% (*n* = 5,253). We further excluded 421 participants whose parents were deceased, divorced, or remarried, given that they were also negatively affected by other determinant parental effects on mental health and behaviors (37). Ultimately, a total of 4,832 samples (89.6%) were incorporated into our analysis, composed of 1,025 BLBC (21.2%), 1,180 FLBC (24.4%), 139 MLBC (2.9%), 1,330 PLBC (27.5%), and 1,158 NLBC (24.0%).

Table 1 presents the socio-demographic characteristics of each group. Overall, the age (mean 13.04, SD 1.28) ranged from 10 to 16 and differed significantly (*p* < 0.001). There was no difference in gender or only-child status but in grade (*p* < 0.001). Also, the groups varied significantly in both household wealth and parental education level. Relatively, BLBC and NLBC, as opposite to MLBC, reported better household finances. Yet, both BLBC and MLBC had lower parental education attainment. Household registration status was also statistically different (*p* < 0.001), with a proportion of urban hukou in NLBC (76.9%) and rural hukou in BLBC (68.2%).

**Table 1:** Sample characteristics of study participants by parental migration status, mean (SD)/*n* (%).

	**BLBC** ***n* = 1,025**	**FLBC** ***n* = 1,180**	**MLBC** ***n* = 139**	**PLBC** ***n* = 1,330**	**NLBC** ***n* = 1,158**	***F* or χ^2^**	***p***
Age, mean (SD)	13.13 (1.26)	13.10 (1.28)	13.10 (1.22)	13.12 (1.28)	12.80 (1.29)	12.926	<0.001
**Gender**, ***n*** **(%)**						3.032	0.552
Men	568 (56.2)	674 (58.1)	84 (60.4)	744 (56.6)	628 (55.0)		
Women	443 (43.8)	487 (41.9)	55 (39.6)	571 (43.4)	513 (45.0)		
**Grade**, ***n*** **(%)**						30.470	<0.001
Grade 5–6	449 (43.8)	486 (41.2)	59 (42.4)	518 (39.0)	573 (49.5)		
Grade 7–8	575 (56.2)	693 (58.8)	80 (57.6)	810 (61.0)	584 (50.5)		
**Household wealth level**, ***n*** **(%)**						23.534	<0.001
Much better off/better off	280 (27.6)	283 (24.2)	31 (22.5)	315 (24.0)	339 (29.8)		
The same	670 (66.1)	770 (65.8)	94 (68.1)	892 (67.9)	731 (64.2)		
Poorer/much poorer	64 (6.3)	117 (10.0)	13 (9.4)	106 (8.1)	68 (6.0)		
Parental education level, *n* (%) *Kruskal–Wallis test						63.718	<0.001
Primary school or lower	126 (13.8)	176 (16.2)	18 (15.0)	179 (14.9)	116 (11.4)		
Middle school	596 (65.4)	647 (59.5)	76 (63.3)	696 (58.0)	518 (50.7)		
High school or above	190 (20.8)	264 (24.3)	26 (21.7)	326 (27.1)	387 (37.9)		
**Only child**, ***n*** **(%)**						8.682	0.070
Yes	335 (32.7)	339 (28.8)	44 (31.7)	408 (30.7)	394 (34.1)		
No	690 (67.3)	840 (71.2)	95 (68.3)	921 (69.3)	763 (65.9)		
**Household registration status**, ***n*** **(%)**						499.060	<0.001
Urban	326 (31.8)	709 (60.1)	58 (41.7)	857 (64.4)	890 (76.9)		
Rural	699 (68.2)	471 (39.9)	81 (58.3)	473 (35.6)	268 (23.1)		

*BLBC, left-behind children with both parents migrating; FLBC, left-behind children with only father migrating; MLBC, left-behind children with only mother migrating; PLBC, previous left-behind children with one or both parents migrating; NLCB, never left-behind children; Data are mean (SD), n/N (%), unless otherwise indicated. Differences in measurement data were assessed using one-way ANOVA, the Chi-squared test, or the Kruskal–Wallis test as appropriate*.

Group differences in SDQ scores are shown in Table 2: BLBC (*p* < 0.001), MLBC (*p* < 0.05) and FLBC (*p* < 0.01) scored significantly higher than NLBC did on emotional symptoms, hyperactivity, and total difficulties. PLBC (*p* < 0.01, *p* < 0.01, and *p* < 0.001, respectively) also had significantly higher scores than NLBC on the same aspects. The scores on prosocial and peer problems showed ittle difference overall. However, regarding the conduct problems, the scores gained by MLBC (*p* < 0.05) and PLBC (*p* < 0.05) went dramatically higher than that of NLBC.

**Table 2:** Group differences in terms of SDQs, mean (SD).

	**BLBC (1)**	**FLBC (2)**	**MLBC (3)**	**PLBC (4)**	**NLBC (5)**	***F***	***p***	***Post hoc***
Emotional symptoms, mean (SD)	3.57 (2.24)	3.42 (2.25)	3.61 (2.22)	3.46 (2.26)	3.06 (2.19)	8.553	<0.001	1>5, 2>5, 3>5, 4>5
Conduct problems, mean (SD)	2.48 (1.64)	2.47 (1.67)	2.69 (1.93)	2.52 (1.69)	2.30 (1.64)	3.656	0.006	3>5, 4>5
Hyperactivity, mean (SD)	3.94 (2.18)	3.95 (2.15)	4.03 (2.17)	4.00 (2.19)	3.62 (2.19)	5.847	<0.001	1>5, 2>5, 3>5, 4>5
Peer problems, mean (SD)	2.65 (1.69)	2.65 (1.69)	2.71 (1.56)	2.58 (1.67)	2.50 (1.69)	1.779	0.130	
Prosocial, mean (SD)	6.95 (2.02)	7.06 (2.06)	6.89 (2.18)	6.97 (2.05)	7.14 (2.05)	1.707	0.145	
Total difficulties score, mean (SD)	12.65 (5.48)	12.49 (5.60)	12.77 (5.38)	12.54 (5.57)	11.47 (5.50)	8.463	<0.001	1>5, 2>5, 3>5, 4>5

*Post hoc indicates the significance of pairwise comparisons in the post-hoc analysis*.

*BLBC, left-behind children with both parents migrating; FLBC, left-behind children with only father migrating; MLBC, left-behind children with only mother migrating; PLBC, previous left-behind children with one or both parents migrating; NLCB, never left-behind children; Data are mean (SD), unless otherwise indicated. Differences in measurement data were assessed using one-way ANOVA and Scheffe's test*.

As illustrated in Table 3, either the smoking rate or drinking rate in BLBC and FLBC (all *p* < 0.05) was considerably higher than that of NLBC, and the same went for PLBC (both *p* < 0.05). Notably, the smoking prevalence among MLBC (*p* < 0.05) with 25.2% more than doubled that of NLBC (11.3%). The drinking rate, as compared to that in NLBC, was also much higher in MLBC, although less significantly. On internet addiction, only MLBC (25.8%) revealed a difference as compared with NLBC (*p* < 0.05), being over two times the prevalence in NLBC (14.5%).

**Table 3:** Risk behaviors by parental migration groups, *n* (%).

	**BLBC (1)**	**FLBC (2)**	**MLBC (3)**	**PLBC (4)**	**NLBC (5)**	**χ^2^**	***P*-value**	***Post-hoc***
**Ever smoking (%)**						40.969	<0.001	1>5, 2>5, 3>5, 4>5
No	839 (81.9)	974 (82.5)	104 (74.8)	1,068 (80.3)	1,026 (88.7)			
Yes	185 (18.1)	206 (17.5)	35 (25.2)	262 (19.7)	131 (11.3)			
**Ever drinking (%)**						22.796	<0.001	1>5, 2>5, 4>5
No	625 (61.0)	730 (61.9)	87 (62.6)	823 (61.9)	803 (69.3)			
Yes	400 (39.0)	450 (38.1)	52 (37.4)	507 (38.1)	355 (30.7)			
**Internet addiction (%)**						12.029	0.017	3>5
No	618 (80.4)	729 (82.5)	72 (74.2)	844 (81.7)	682 (85.5)			
Yes	151 (19.6)	155 (17.5)	25 (25.8)	189 (18.3)	116 (14.5)			

*Post hoc indicates the significance of pairwise comparisons in the post-hoc analysis*.

*BLBC, left-behind children with both parents migrating; FLBC, left-behind children with only father migrating; MLBC, left-behind children with only mother migrating; PLBC, previous left-behind children with one or both parents migrating; NLCB, never left-behind children; Data are n/N (%), unless otherwise indicated. Differences in measurement were assessed using the Chi-squared test*.

Table 4 renders the regression results for comparing the SDQ scales by different parental migration and children's sociodemographics. Unsurprisingly, after adjusting sociodemographic variables (age, grade, household wealth level, parental education level, and household registration status), BLBC (*p* < 0.001), FLBC (*p* < 0.01), and MLBC (*p* < 0.05) correlated to emotional symptoms significantly compared to NLBC, together with the conduct problems. In particular, the above issues were strongly linked to MLBC along with BLBC. It was also noted that PLBC showed a significant association with either emotional problems or conduct issues or hyperactivity. Overall, a correlation to total mental difficulties by comparison of NLBC was seen in all other groups and was remarkably stronger in MLBC [β = 1.19, 95% CI (0.13, 2.26), *p* < 0.05]. Besides, the results also showed that girls, children who reported to be from lower-income households, and those with lower parental education were significantly related to overall mental difficulties as well, as compared to their male counterparts.

**Table 4:** Linear regression analysis for SDQ by parental migration groups and demographic characteristic, β (95% CI).

	**Emotional symptoms**	**Conduct problems**	**Hyperactivity**	**Total difficulties score**
**Parental migration status (ref: NLBC)**				
BLBC	0.47 (0.26, 0.68)***	0.23 (0.07, 0.39)**	0.26 (0.05, 0.46)*	1.13 (0.60, 1.65)***
FLBC	0.27 (0.07, 0.46)**	0.17 (0.03, 0.32)*	0.19 (0.01, 0.38)*	0.79 (0.30, 1.28)**
MLBC	0.48 (0.06, 0.91)*	0.41 (0.09, 0.72)*	0.28 (−0.13, 0.69)	1.19 (0.13, 2.26)*
PLBC	0.33 (0.14, 0.51)**	0.25 (0.11, 0.39)**	0.34 (0.16, 0.52)***	1.00 (0.53, 1.47)***
**Gender (ref: male)**				
Women	0.63 (0.49, 0.76)***	−0.15 (−0.25, −0.05)**	0.23 (0.09, 0.36)**	0.48 (0.14, 0.82)**
**Grade (ref: grade 5–6)**				
Grade 7–8	0.07 (−0.07, 0.20)	0.08 (−0.02, 0.19)	0.51 (0.38, 0.64)***	0.35 (0.01, 0.69)*
**Household wealth level (ref: much better off/better off)**				
The same	0.03 (−0.13, 0.18)	−0.08 (−0.20, 0.03)	0.26 (0.11, 0.41)**	0.28 (−0.11, 0.67)
Poorer/much poorer	0.81 (0.52, 1.09)***	0.35 (0.14, 0.57)**	0.70 (0.43, 0.98)***	2.58 (1.87, 3.30)***
**Parental education level (ref: high school or above)**				
Primary school or lower	0.13 (−0.10, 0.36)	0.13 (−0.04, 0.31)	0.52 (0.30, 0.75)***	1.05 (0.48, 1.62)***
Middle school	0.04 (−0.12, 0.20)	0.01 (−0.12, 0.12)	0.29 (0.14, 0.45)***	0.40 (0, 0.80)
**Only child (ref: yes)**				
No	0.04 (−0.10, 0.19)	−0.01 (−0.12, 0.10)	0.03 (−0.11, 0.17)	0.01 (−0.36, 0.37)
**Household registration status (ref: urban)**				
Rural	−0.01 (−0.16, 0.13)	−0.11 (−0.21, 0.01)	0.08 (−0.06, 0.22)	0.01 (−0.36, 0.37)

*p < 0.05.

**p < 0.01.

***p < 0.001.

*BLBC, left-behind children with both parents migrating; FLBC, left-behind children with only father migrating; MLBC, left-behind children with only mother migrating; PLBC, previous left-behind children with one or both parents migrating; NLCB, never left-behind children; Multiple linear regression and logistic regression were employed for SDQ scores and demographic characteristic, as appropriate. Data are β or odds ratios (ORs) with 95% confidence intervals (CIs), unless otherwise indicated*.

According to logistic regression analysis in Table 5, each type of parental migration also showed a conspicuous relevance to the risk behaviors among children. By contrast with NLBC, all LBC, as well as PLBC, showed higher odds of smoking. In particular, MLBC were more likely [OR = 2.31, 95% CI (1.45, 3.69) *p* < 0.001] to smoke than NLBC. As for the drinking problem, there remained a significant correlation among BLBC, FLBC, and PLBC, except MLBC. After controlling other variables, BLBC and MLBC significantly correlated with internet addiction, and the latter [OR = 2.15, 95% CI (1.24, 3.72), *p* < 0.01] were even more likely than NLBC to suffer. Additionally, there existed a gender gap in smoking, with boys at higher risk. Rural children were more likely to exhibit smoking problems than the urban ones, and children from urban were at higher risk than rural ones for internet addiction.

**Table 5:** Logistic regression analysis for risk behaviors by parental migration groups and demographic characteristics, OR (95% CI).

	**Ever smoking**	**Ever drinking**	**Internet addiction**
**Parental migration status (ref: NLBC)**			
BLBC	1.66 (1.27, 2.18)***	1.35 (1.10, 1.65)**	1.50 (1.11, 2.02)**
FLBC	1.49 (1.15, 1.93)**	1.26 (1.05, 1.52)*	1.07 (0.80, 1.42)
MLBC	2.31 (1.45, 3.69)***	1.12 (0.75, 1.68)	2.15 (1.24, 3.72)**
PLBC	1.80 (1.41, 2.30)***	1.28 (1.07, 1.53)**	1.22 (0.93, 1.60)
**Gender (ref: male)**			
Women	0.64 (0.54, 0.76)***	0.93 (0.82, 1.06)	0.83 (0.68, 1.00)
**Grade (ref: grade 5–6)**			
Grade 7–8	1.63 (1.37, 1.94)***	1.58 (1.39, 1.81)***	2.65 (2.12, 3.31)***
**Household wealth level (ref: much better off/better off)**			
The same	1.02 (0.84, 1.24)	0.94 (0.81, 1.09)	0.78 (0.63, 0.97)*
Poorer/much poorer	1.25 (0.90, 1.73)	0.86 (0.66, 1.13)	1.29 (0.90, 1.85)
**Parental education level (ref: high school or above)**			
Primary school or lower	1.33 (1.02, 1.75)*	1.07 (0.86, 1.34)	1.42 (1.03, 1.96)*
Middle school	1.10 (0.90, 1.34)	1.17 (1.01, 1.36)*	1.31 (1.04, 1.65)*
**Only child (ref: yes)**			
No	1.09 (0.91, 1.31)	0.97 (0.84, 1.11)	1.09 (0.88, 1.34)
**Household registration status (ref: urban)**			
Rural	1.19 (1.01, 1.42)*	1.14 (0.99, 1.31)	0.67 (0.55, 0.83)***

*p < 0.05.

**p < 0.01.

***p < 0.001.

*BLBC, left-behind children with both parents migrating; FLBC, left-behind children with only father migrating; MLBC, left-behind children with only mother migrating; PLBC, previous left-behind children with one or both parents migrating; NLCB, never left-behind children; Logistic regression were employed for ever smoking, ever drinking, internet addiction and demographic characteristic, as appropriate. Data are odds ratios (ORs) with 95% confidence intervals (CIs), unless otherwise indicated*.

## Discussion

Overall, our findings manifested a compelling presence of mental health concerns, including emotional symptoms, conduct problems, and total difficulties among all types of current LBC (BLBC, MLBC, and FLBC) and even among PLBC. Also, the risk of smoking and drinking was pervasive across these groups. However, the varying parental migrations contributed to different correlations to the mental and behavioral troubles among LBC within comparison to NLBC. The MLBC, in particular, were at greater risk of mental health difficulties and health-related behaviors concerning smoking and internet addiction.

For current LBC, any type of them was characterized by parental absence due to migration. The separation between parents and children could broadly affect their family structure, child-caring arrangements, and parent–child relationships (38). This also accounted for how migrant parents influenced a broad range of health outcomes among LBC (39, 40). As empirical evidence has long acknowledged, we suggested that the parental migration affected BLBC's mental health extensively, accounting for both internalizing (emotional symptoms) and externalizing problems (conduct problems and hyperactivity) (16, 41). These risks agreed well with the attachment theory, a perspective of child development (42). In the absence of parents, BLBC tended to have an insecure parent–child attachment, which could negatively affect their social and emotional development and contribute to internalizing or externalizing problems (43, 44). Yet, we further underscored that these consequences significantly existed among FLBC similarly, with the additional support of the statement that paternal attachment exerts a critical effect on child mental health (45). Partially consistent with most suggestions, our results indeed captured that the migrant father served as an economic contributor and might partly buffer their negative health implication behind (46, 47). However, we also lent another support to the limited research focusing on this largely overlooked health aspect and psychological wellbeing (48) and highlighted that the influence on the health of FLBC called for greater attention still.

Even more alarmingly, despite being a minority within LBC, MLBC presented a stronger correlation to emotional and conduct problems with higher scores, implying the worse affected mental health. This embodied a barely discussed but serious consequence among this small but more vulnerable group. The MLBC, by contrast, was not just being less socioeconomically advantaged but also going through the interruption of their relatively strong attachment to mothers (44, 49). This, as the attachment theory goes, could compromise their mental and emotional development more severely, accounting for more worrying mental consequences (50). In fact, drinking or smoking problems were found among MLBC, BLBC, and FLBC with higher prevalence. As the well-established evidence suggested (51), this might be due to parental knowledge and weak supervision toward children and the low quality of the parent–child relationship with absent parents. Additionally, the combination of a greater vulnerability in mental health that correlates to substance abuse and the potential direct effect of parental (often fathers among MLBC) smoking behavior (51–53) might account for the rather alarming rate of smoking among MLBC as well. Besides, the problematic mother–adolescent communication that had a worse influence on their substance abuse might be another explanation, as we addressed in a previous study (54). Additionally, given that, in China, parenting remains mainly the responsibility of the mother, culturally (28), MLBC might accordingly fall at more disadvantage in preventing these risk behaviors when their mother migrates and exhibit a higher prevalence of substance use. In addition, in line with a longitudinal study confirming the absent mother as the risk for children's addiction to the internet (55), we also suggested a robust association between internet addiction and MLBC. Similarly, this is largely rooted in the dearth of mother-child attachment with less maternal emotional warmth and paternal neglectful parenting (25, 30, 34, 56). Overall, we underscored that, though small in proportion among LBC, MLBC nevertheless were doubly disadvantaged with worse mental and behavioral health. The findings not only echoed the existing analysis of studies limited to rural areas (19, 57) but further broadened the evidence on children in urban areas. However, with a small sample size of MLBC, caution must be applied. More analysis of robustness in a larger sample of MLBC is still needed.

Other than current LBC, our findings also highlighted the mental difficulties and risk behaviors among PLBC. Adding weight to the earlier study (21), we suggested that the adverse influence of parental migration on mental health and behavioral aspects might remain constant for PLBC regardless of no longer being left behind. For these children, aside from the experience of separation from parent(s), there existed a new challenge of readapting changes of caregivers and family structure, which might also pose troubles (39). The findings also gave impetus for a longitudinal study to confirm and for future research to distinguish between PLBC (previous MLBC, FLBC, and BLBC) for further insights into the difference in parental roles.

Several limitations in this study need to be noticed. First, the associations established in this observational study cannot be interpreted as causality despite the literature support. Second, we failed to specify the parenting practices in mental and behavioral caretaking from either caregivers or parents, and their risk behaviors also might be confounding factors. Third, children's self-reporting might introduce recalling bias. Given these limitations, a comprehensive collection of questionnaires based on teachers, parents, and caregivers is being conducted in our follow-up study. Also, the classification of parental migration by measuring the duration and frequency of parental migration in detail is another priority in our following survey. Finally, the analyzed sample was only from one area in western Anhui Province, the second largest province with an out-migrant population in China (58), yet the nationwide generalizability was still limited.

Still, this study provided a more comprehensive picture of the mental and behavioral concerns among LBC differing from parental migration. This carried some implications for intervention and policy-making concerning LBC. First, we provided insights into the underlying heterogeneity of LBC in parental migration to capture the more vulnerable group. Also, the study contributed to a better understanding of the specific health risk among children left by different parents, enabling migrant parents to weigh options for the minimal impact on children. Besides, we added evidence for the necessity of continuous concern for LBC and the children who previously experienced being left. Overall, differentiating the LBC is needed not only in developing required research but also in ensuring that the tailored intervention and policies are in line with the needs and relevance to a specific migrant family's context.

Encouragingly, as one glimmer of hope to the vulnerable, China is rolling out practical measures to improve the care and service for LBC as well as children in difficulty. As one step, many local governments have equipped with a “children director” for each village and a “children supervisor” for each township to guarantee timely discovery and protection. This study expanded the existing findings and emphasized different types of LBC to capture more specifically vulnerable ones. Our results also implied that the significant but different proportion of rural and urban LBC called for continuing and specific efforts to support services for access to their caretaking in both settings. Additionally, policy response so far might further need to focus on their health service equity as well. There is a need to align MLBC with priority and gain attention to the inclusion of PLBC. Concerning FLBC, who account for a relatively large proportion of LBC within the current context of migration in China (2), there would seem to be a definite need for supporting FLBC for their poor mental health with policy response in turn. Most importantly, the more effective interventions and policies targeting different types of LBC entail more in-depth studies to build robust evidence, which is the key to progress.

## Conclusions

This study illustrated the presence of psychological concerns and risk behaviors among LBC differing from parental migration to explore the specific impacts of each migrant parent. We accentuated that either parent's migration could give rise to a pervasive risk for poor mental and behavioral health and that MLBC were particularly vulnerable. Additionally, PLBC with experience of being left behind remained doubly disadvantaged than their counterparts. Collectively, we highlighted that the impacts of each migrant parent on children's general health all matter but also vary. Regarding the future intervention for LBC, there is a need to align MLBC with priority and to assure attention to the inclusion of PLBC. Also, the follow-up research needs to be definite and to extend these findings to a more detailed status of parental migration to clarify.

## Data Availability Statement

The raw data supporting the conclusions of this article will be made available by the authors, without undue reservation.

## Ethics Statement

The studies involving human participants were reviewed and approved by Ethics Committee of Zhejiang University. Written informed consent to participate in this study was provided by the participants' legal guardian/next of kin.

## Author Contributions

FW, LL, and XZ contributed to the design of the study. JL, GZ, and HA performed the statistical analysis. JX and MW were responsible for the survey supervision. FW, LL, XZ, and HA wrote and reviewed the manuscript. All authors agreed to be accountable for the content of the work.

## Funding

This work was supported by the China Postdoctoral Science Foundation (Grant No. 2020M671777), Zhejiang University Zijin Talent Project of China, and the National Social Science Fund of China (Grant No. 21CRK015). The funding institution had no role in the study design, the collection, analysis, interpretation of the data, and the decision to publish or preparation of the manuscript.

## Conflict of Interest

The authors declare that the research was conducted in the absence of any commercial or financial relationships that could be construed as a potential conflict of interest.

## Publisher's Note

All claims expressed in this article are solely those of the authors and do not necessarily represent those of their affiliated organizations, or those of the publisher, the editors and the reviewers. Any product that may be evaluated in this article, or claim that may be made by its manufacturer, is not guaranteed or endorsed by the publisher.
